# American Academy of Dental Science

**Published:** 1901-11

**Authors:** 

**Affiliations:** American Academy of Dental Science


### Reports of Society Meetings.

### AMERICAN ACADEMY OF DENTAL SCIENCE.

The regular monthly meeting of the American Academy of Dental Science was held at Young's Hotel, Boston, on Wednesday evening, May 1, 1901, at six o'clock.

In the absence of both the President and the Vice-President, Dr. Thomas Fillebrown was chosen as the presiding officer.

Two papers were presented, one by B. Holly Smith, M.D., D.D.S., of Baltimore, Md., entitled "Some Æsthetic Considerations in the Treatment of Teeth in the Incisal Region;" the other by L. S. Chilcott, D.D.S., of Bangor, Me., entitled "The Use of Silver-Foil in Dentistry."

*Dr. Fillebrown.*—I am called to the duties of this office to-night very unexpectedly, but I am happy to say the duties of the presiding officer of the Academy are very easy.

It is customary, I believe, in introducing a speaker to pay him many compliments; but I am happy to introduce a man to-night who does not need it, because he will before the evening is out establish his right and title to all the compliments we can give him. It gives me great pleasure to introduce to you, as one of the essayists of the evening, Professor B. Holly Smith, of Baltimore.

(For Dr. Smith's paper, see page 737.)

DISCUSSION.

*Dr. B. Holly Smith* (after his paper, referring to one of the models).—This patient had had appliances made to move the cuspid into line which were not effective. I thought at first the movement might be accomplished by using the bicuspids and molar on the opposite side as abutment. It was discovered soon that the cuspid was more firmly fixed than the opposing teeth to which the appliance was attached. I then included (as shown by the model) the bicuspids and molar on the same side as the offending cuspid. This was set with cement, the screws aiding in fixation. In about a week the young man came in with the tooth in position, as shown in the model.

*Dr. Fillebrown.*—It gives me great pleasure to add my personal endorsement to what the Professor has said to-night, with hardly an exception. The subject is now open for discussion.

*Dr. Taft.*—Will the essayist please state again what purpose these little screws on the side are intended to serve?

*Dr. Smith.*—It was found that the bridge was unseated; that is to say, the bands were unseated by the pressure, and after it once became loose it was determined to put those screws in so that a bearing or impact would be had on the bevel,—on the approximate bevel of the teeth,—and thus add security to the bridge. With the screws in position and without cement it was impossible to remove the bridge, but it was simply to give anchorage and steadfastness to the appliance.

*Dr. Fillebrown.*—If the Chair understands the gentleman, the screws pass in between the necks of the teeth, up near the gum, and, consequently, going in between, they cannot get past the bulge of the teeth.

*Dr. Smith.*—If you will allow me, Mr. President, I would like to say that I feel almost sorry that I brought these models. I hope the trend of the discussion will not in any sense be dwarfed by a consideration of this small exhibition of orthodontia.

*Dr. Fillebrown.*—There is not a man here who is not brimful. I know Dr. Andrews has something worth hearing.

*Dr. Andrews.*—Mr. President, I am always glad to hear and welcome Dr. Smith. What he has to say is well worth hearing, and the evening's paper has been particularly interesting to me, especially that point in regard to improving the appearance of the teeth by the simple method of shaping them. Perhaps every Fellow of the Academy has given more or less attention to this class of work and appreciates its value. I know of nothing that gives the same satisfaction to the patient when the operation is performed in an artistic manner. Every suggestion in the paper is worthy of our close attention, and there is certainly nothing that I can suggest to better his conclusions. One suggestion which seems to me particularly interesting and valuable is in regard to putting on bridges without the unsightly gold abutments, and putting on crowns without the gold band showing at the neck of the tooth. The method which I have used is somewhat different from the one which he described. I shape a band about two-thirds around the tooth, when I fit a crown similar to the method suggested by Dr. Litch some years ago. In making bridges, where I find it necessary to use a cuspid or a bicuspid tooth for an abutment I use the method devised by Dr. Marshall, of this city,—cutting a groove across the back of the tooth and down each side of it. Into this groove a U-shaped piece of platinum and iridium screw wire is fitted. Over this I burnish a piece of thin gold plate and solder the wire to the burnished plate. This cap of gold, with its wire, is used for the abutment of the cuspid or bicuspid tooth, and, soldered to the bridge, becomes its forward abutment. Cemented in place, this shows no gold whatever in front and adds very much to the beauty of work of this kind.

The matter of regulating teeth in adult life, I confess, is somewhat surprising to me. I have never dared to do it, or at least in only a few favorable cases. I have feared that the process would not form around these teeth, especially where there were any pathological indications. Dr. Smith's success inspires me with a new courage; I know that I shall profit by what he has suggested.

The paper is an inspiration to him whose mission it is to beautify the mouth. I do not think that there is anything else that I can say; the essayist has covered the ground so completely that there is little to be said, except in commendation.

*Dr. Piper.*—If it is in order, I would like to ask Dr. Smith a question with regard to this case of central incisors. How does he retain them? I have a case almost exactly like it.

*Dr. Smith.*—I should not hesitate at all to apply a backing to the lingual surface of those teeth, and, using a thread that we have in the little Howe set of instruments, I should set with cement and screw this thread into position. I would not hesitate at all to find anchorage in the dentine of these teeth and put on a backing to hold them permanently, not assuming that they would stay in position. I have no idea that they will. This is a case in process of regulating. But to see the woman when she came into the office! Her mouth was so unsightly that it seemed an eloquent appeal—if ugliness can be appealing—for remedy, for help. And when she told me she had consulted three or four dentists and asked if anything could be done, I felt sorry that such a state of helplessness was possible. Anything would be better than the condition we have at present. And, of course, your own resourcefulness would suggest something to hold those teeth in position.

*Dr. Piper.*—I would like to state that that is just what I did.

*Dr. Fillebrown.*—If you will permit a departure from parliamentary rules, and allow the Chair just a word in regard to this process, I will state that I have verified the wisdom and benefits of that operation in at least half a dozen cases. One marked case, and I think about the first one I had, was some ten years ago. A patient was referred to me with upper central incisors just like this, very seriously affected by Riggs disease. I put on a rubber band and drew them together, which was quite easy to do. Then I drilled a hole in each tooth just in front of the cingulum and made a little staple (I did not trouble for any screws) that just reached into the holes that I drilled, and hooked it in so that the spring of the teeth would hold it. I filled with amalgam, and the teeth have remained in position. Had I known, I would have brought the model of them. They are now in perfect position. Although they were very seriously affected with Riggs disease, there is scarcely a trace of it around the teeth at the present time.

I think it a most admirable way to do, perfectly reasonable, and it may be made entirely successful. In one case four teeth were in bad positions from Riggs disease. I brought them into place and struck a backing of thin gold, and, putting in pins, cemented them, and thus held them in place for a long time.

*Dr. Werner.*—The paper is full of suggestions, and the Fellows of the Academy appreciate them as nicely stated. Often we should abolish the use of pure gold in the incisor region, substituting platinum and gold, if we have to use a metal filing.

Then the ground inlays in distinction from the baked ones must be very apparent to all of us.

Then the motto in his appointment book,—to it I wish to add Lowell's quotation, that "not failure, but low aim, is crime." Let us think of it often. What a satisfaction to do a real good thing, for which your patient will be thankful to you forever! We should have that high aim,—*i.e.*, to do the nicest thing for the longest time.

And as to the gold crowns on bicuspids or molars, to me they are ugly-looking things. How much better to have a carved tooth that looks like the other teeth.

There are many other points that might be favorably commented upon. We should never do a merely mechanical thing, at the expense of all laws of æsthetics and nicety. There is a vulgarity in the show of gold in or about the teeth.

It is difficult to match cements for teeth that have had the rubber dam on. Hence I make it a point to mix the cement to the color of the tooth in its moist condition, and not as the tooth is after the rubber dam has been on for some time.

*Dr. Fillebrown.*—No discussion is complete without a word from Dr. Meriam, when he is present.

*Dr. Meriam.*—Mr. President, I think we can all say with—I think it was one of our Boston poets:

> "And there's a nice youngster, of excellent pith;
> Fate could not conceal him by naming him Smith."

Through all this paper we can see the man behind it. To my mind, to-day as much as ever, perhaps I will say more than ever, with the multiplication of our appliances and everything of that kind, there still remains this, that you have got to have the man, and the purpose of the man directing all these things, to get the best results. That which is introduced as an adjunct may without judgment and care be the unmaking of a man. Your cements and amalgams, if the man has not the purpose to keep the work up and his practice up, can be the means of making him lazy, of shrinking from the objections of his patients, so that he does not devise and does not do the best things. That, I think, is perfectly true. And so it is a refreshing thing to think that a practitioner starts out each year with a high resolve.

There is another thing that the essayist spoke of, the crowning of teeth, and I think what he says is very true where we decide upon the amputation of a tooth. But when it comes to the tooth that has decided for itself, being broken and irregular, I do not know of any way of caring for this tooth except by bands.

There is another question, and I have done something at it myself, but not much. We have colored gold and made it fairly successful for fillings. Now, why should we not be working along the lines of colored gold for crowns? We can take alloys as low as eighteen in the mouth, and that gives us six grains in a pennyweight to work on for the change of color. And while platinum makes gold hard, we can, perhaps, in working with some other metals, metals that may not be in use in dentistry to-day, work along and get a plate for crown-work that is as successful as the colored gold that we now use for fillings. Of course, the jewellers have all sorts of colored golds, and there are recipes for making and treating them. They are not for us, but they may furnish a suggestion for the next original worker.

With regard to the straightening of teeth in old age, the correction of irregularities, I referred to a case, I think, at the time Dr. Decker was here where I lifted out the whole six front teeth of a lady who was considerably over forty.

I do not think there is much more that I can add, excepting the pleasure that we all feel in New England in having a representative of the South, or the nearly South, among us this evening.

*Dr. Clapp.*—Mr. President, I think if there is any one thing that we admire it is an exhibition of courage, and if there is anything in this world that requires courage, it is for a busy dentist to attempt just this sort of work Dr. Smith has shown us.

It will be necessary for the correct understanding of his paper to have the models reproduced, and I understand it will assist our Editor considerably if he has authority to expend money in the reproduction of these models, and I therefore move that the Academy appropriate a sufficient sum for the illustration of the paper.

So voted.

*Dr. Fillebrown.*—There is at least one other member here whose conscious or unconscious cerebration always produces something worth while, and if we do not get the benefit of it, it will be just so much clear loss. I suggest that Professor Brackett tell us what he has been thinking about.

*Dr. Brackett.*—Mr. President, one or two things I will say: first, to express the gratification that we all have felt in listening to the paper; second, to set up something which the essayist did not mean, and then object to that something.

In the sense that the essayist intended, I suppose I am in accord with his sentiment that the patient should be as "clay in the hands of the potter." In another sense, which he probably did not mean, I should object to that. I think it is very profitable for the practitioner to listen to the patient's suggestions. I remember our friend, Dr. Henry J. Bigelow, the surgeon whose attainments commanded the admiration of the surgical world, saying that every man knows his own geography best. It is true that patients, as they dwell upon their own embarrassments, their own sufferings, or their own needs, do gain in some particulars in multitudes of instances a better acquaintance with their defects and their needs than does the practitioner in a briefer inspection. Perhaps I am not up to the standard of the average dentist in attainment or alertness to see, but I have been many times very greatly profited by hearing that which the patient suggested. I have sometimes thought, without ever having had any positive statement to that effect, that the custom which I believe obtains in medical practice in consultations, of letting the younger and more inexperienced practitioners speak first, was of advantage to the older and presumably abler men who spoke after the suggestions of the supposedly less competent men had been heard.

So, without any spirit of criticising that which the essayist did not mean, I think there is in this which I have expressed that which has been profitable to me in my practice.

*Dr. Werner.*—I would like to ask the essayist whether he retains these regulating fixtures with cement or gutta-percha while the regulating is going on?

*Dr. Smith.*—It is set with cement usually. The only appliance that has been presented was set with cement.

*Dr. Werner.*—I should think it would be absolutely necessary: it would be wholly unsafe without.

*Dr. Smith.*—Mr. President, I only want to say that I cordially appreciate your kind words and am touched with a sense of your brotherly love in saying them. I have had nothing but admiration for this august and learned assembly since I have known of its work. I have read your proceedings, and have felt that your influence was great in shaping the practice and morals of the profession, and I almost feared to present the subject-matter of this paper before your body. When we know the history of the organization, when we know what you have been doing, when we know that you have had men of ability, of reputation, before you, and know the class of work which you have done, it does seem a little tame to go back and rake up such a trite matter as I have brought to you.

And still I confess to you that I had it on my conscience that what has been said should be said. I have had it driven home to me quite recently. I had my boys come home for their Easter holiday, and they brought with them four students in a preparatory institution. One of them was the son of a physician, a specialist, a man of reputation and ability, of position and wealth. I could but notice that his expression was marred by the presence on the left lateral incisor of a gold crown. The first bicuspid on the right was crowned with gold, and two extensive contoured gold fillings were in his mouth. He was a Baltimorean. Another boy was from New York. His mouth was quite as unsightly, with an altogether unnecessary display of metal, one gold crown and large contour fillings.

Last week we had an assembly of Congregational ministers in our town, and two of them stopped at my house, one of them a charming man, a graduate of a university, a man whose very smile, if you could catch it without the marred effect of the presence of gold, would have been most contagious and most delightful. Even my wife, who perhaps might be placed in the position of a layman, one who does not think so much of these things, was struck with the revulsion which I could not but show when he smiled. He had a gold crown on the right first bicuspid and large gold fillings. He had no beard nor moustache, a clean, nice man, who took the utmost care of his person, dress, and manners, and still by the hand of a brother dentist he was marred. Should this thing exist?

I visited some folks here in Boston. I did not expect to find anything like that in Boston, but I have been around a little bit to-day, and I was introduced to a gentleman of position, of learning, a graduate of your University, a man of culture and training. What did I see in his mouth! He must have been in the hands of a dentist—not here, I hope.

These things, gentlemen, stare us in the face. Is it too late to go back and say that this practice, which I contend is barbarous, must be revolutionized? Is it too early to say we have outlived such practice—we do not do it now? Maybe *we* do not. Some people *do*. It is our business to see that they do not. Let us see if we cannot stop it.

*Dr. Fillebrown.*—I am sure, if I was situated differently this evening, I should be glad to occupy some minutes; but the time is passing, and we have others to hear from, and perhaps we had better entertain a motion that we now pass the subject.

Subject passed.

*Dr. Fillebrown.*—Ages ago, I believe, there was a company of men that started with their faces towards the East, and they were following the star in the East. To-night, I am happy to say, the conditions are reversed, and the star from the East has come to us. I am happy to introduce to you Dr. Chilcott, of Bangor, a member of the Board of Registration of the State of Maine, who will have a word for us on the use of silver-foil in dentistry.

(For Dr. Chilcott's paper, see page 690.)

DISCUSSION.

*Dr. Chilcott.*—Mr. President and Fellows of the Academy, when I was considering this paper, I hoped I really had something to say, but when I tried to put it on paper in my homely way, I was chagrined to find that, after all, there was not much of it.

I have asked permission, and obtained it, from the Chairman of the Executive Committee to say a word about cuspidors. You know, if your experience has been anything like mine, that it is not easy to find a sightly, nice, clean cuspidor, one that will remain so, even with care, that you can use in your office. I nagged our local crockery man until I was pretty well ashamed of it, although he took it very pleasantly. He had my order on his list for two or three years. And finally he said it was no use; he knew what I wanted, but he could not get it, as it was not made. In looking about I found some glass rose-bowls, like the one I have here, and am using one of them in which I keep a little clean water. I should like it better if it had a wider rim, but that is all there was on it. The comments which have been passed on it by people of refinement have been decidedly pleasing.

*Dr. Taft.*—How do you place it at the chair?

*Dr. Chilcott.*—It will go in the bracket at the side of the chair which is made for that purpose. A White's bracket is the one I happen to be using; it goes all right in that. The bowl is rinsed out when the patient leaves the chair and returned to its place perfectly clean.

*Dr. Meriam.*—It is, of course, made by some glass manufacturer?

*Dr. Chilcott.*—Yes, they are imported goods, being of a Bohemian make, but the glass-blowers are very obstinate, and while they will make a million all right enough, they will not take a small order.

*Dr. Fillebrown.*—The subject is now open for your consideration, remarks, criticisms, queries, or otherwise.

*Dr. Brackett.*—Mr. President, I would like to ask three questions: first, Where is this silver-foil obtainable? second, Is it feasible to have it of greater thickness, and, if so, is that an advantage or a disadvantage? third, What advantages does it possess over tin-foil?

*Dr. Chilcott.*—The only place I know of where it can be obtained is from Hynson, Westcott & Co., Baltimore.

I do not know why it would not be practical. I wrote to them and asked them, in filling an order, if they would not send me some that was heavier. I got no reply; the foil all came that thickness.

I think in many ways it has an advantage over tin-foil, for the reason that it oxidizes more readily, and that for the filling of pulp-canals it can be carried to the apex with less irritation. I have filled a good many teeth with the non-cohesive gold-foil, first filling the cavity partly full of tin-foil, but I do not know that this in those cases would have worked any better.

*Dr. Taft.*—I would like to ask the essayist what the foil is generally used for as made in this way?

*Dr. Chilcott.*—This foil was designed as a dressing for wounds, as I understand it, that were considered to be surgically clean; instead of covering them up with a pad of cotton or the surgeon's gauze, they put a few layers of this over the wound, and this young student at the medical college says they have most excellent results with it, for it certainly has a germicidal effect, and they get an adhesion by first intention with this oftener than with other methods.

*Dr. Werner.*—Does your experience show you that it does discolor tooth-substance?

*Dr. Chilcott.*—I have seen it where it appeared to discolor, but not in all cases.

*Dr. Werner.*—Does it infiltrate the tooth? Does it discolor the tooth itself?

*Dr. Chilcott.*—I have never used it where I would have a good chance to determine that. I have used it in soft, porous teeth, but they might possibly show a discoloration from something else. The discoloration I have seen in those cases I attributed to the silver-foil. I never had occasion to take any of it out, so I cannot tell you how far it penetrated the dentine.

*Dr. Werner.*—You know tin does not discolor tooth-substance, and we get the same germicidal effect, which is very likely due to the oxidation.

*Dr. Chilcott.*—I should feel that this would be more likely to discolor than tin. It certainly is more easily oxidized. I have seen tin placed in a tooth under a gold filling,—I have done it myself,—and it appeared to stain the tooth badly. Perhaps it did not discolor the tooth, but it would make the tooth look very dark.

*Dr. Banfield.*—Mr. President, I think it is wise for us to be cautious how we use any substance that will discolor teeth, particularly the front teeth, for, as I understand it, discolorations can be bleached when caused by vegetable matter, but not from metallic substances.

I will ask the essayist if he has seen any discoloration from the use of this silver similar to that caused by silver nitrate?

*Dr. Chilcott.*—No, not at all, so far as I have yet learned. It has not been in any of the teeth of my patients a great many months. But with the nitrate of silver you can see discoloration from it right off.

*Dr. Meriam.*—Is this lighter or heavier than the gold-leaf? I think it is about the same thickness. I think they advertise a gold and a silver leaf. I have occasionally found the greatest help and co-operation by going to smaller places where there was a man doing his own work. I remember that some years ago I was giving directions to Mr. Schmidt, in New York, and I noticed a gentleman there giving instructions to him in the making of a brace, and after he was through I spoke to him about one of my children wearing a similar brace. We talked on for some time, and he nodded his head towards Schmidt and said, "A good workman." And I said, "Yes, with the advantage of seeing him work." And he said, "Yes, that decidedly. I like to have my instruments made, if I can, where I can see my workman, and not buy them from a clerk at the shops." I think where the thing is nicely made and polished, and as attractive as it can be made to the eye in form, there is a natural feeling of the salesman, How can any one ask for anything better? But there may be some little difference in detail. It may be a matter of measurement, or the position in which one likes to stand at the chair, that makes it desirable to have that form of instrument; and for that kind of a thing the individual workman, if he take an interest, is a great help. In this making of foil, I have no doubt that with the co-operation of some old refiner we might get forms of gold that we never could get where we have to go to a clerk and it goes through two or three hands before it gets to the man who knows about it. If I were a manufacturer, I should keep my face towards my customer and keep the workman out of sight. I think it would be the natural tendency of business and trade. But as a practitioner, I like to know the workman.

Many of the men in Boston ten years ago took up the practice of going right to the shop and having the workmen make the instruments as they directed. I think they not only got their instruments, but they attained a larger intellectual development and a larger freedom, for any one who is working in this way, if he will secure the friendship of a good workman who knows about these things, he will give him with the greatest pleasure points in the matter.

It is the constant running out after something new and drawing it in that has made dentistry what it is. The growth of things is in keeping in touch with things outside of our profession as well as *in* it. And I think the essayist of the evening has done that, while I do not myself see the advantage of this over tin in any way. I remember some years ago I had very light tin made, thinking that I might get something better, but I found it was not. I think he may find that heavier foil than this will work. A man taking up a new thing like this and working it along, is working along a track that very likely will connect with something else, and in that way increase our resources and bring something of value.

Subject passed.

*Dr. Brackett.*—Under incidents of practice I wish to speak informally of the case I mentioned a month ago, where there was a question about the resection of the alveolus of the upper jaw that was projecting overmuch. It is intended to publish the case, but inasmuch as I mentioned it, I will say that the operation was very nicely performed by Dr. W. R. Howard, the dentist, assisted by Dr. R. E. Darrah, a general surgeon. An incision was made along the lower border of the alveoli from the centre of the cuspid socket to cuspid socket; the soft tissues, including the periosteum, were pushed back, and with sharp, bevelled bone-forceps there was removed the redundant portion of the alveolar process, a piece of bone very nearly a quarter of an inch in thickness extending from cuspid to cuspid. The remaining portion of the alveolus was dressed smoothly. Superabundant gum-tissue was cut back, leaving smooth edges of gum, which were neatly coaptated with a few catgut sutures, with the result that there has been perfect healing under the influence of the careful antiseptic treatment that was adopted throughout. Upon Monday of this week impressions were taken for the making of artificial teeth with the embarrassment of the projecting process entirely removed. The result is a decided success.

*Dr. Baker.*—I would like to ask Dr. Brackett, Would not the same result have been obtained if the anterior portion of the alveolar process had been excised? Would not that have been much easier?

*Dr. Brackett.*—That is just what was done, the excision of the anterior portion of the alveolar process, the actual cutting away of that portion of it which was not desired and which made the deformity.

*Dr. Baker.*—I understood from what you said that the bone behind the palatal surface of the teeth was excised and the alveolar process forced back.

*Dr. Brackett.*—Oh, no! An incision was made through the gum from cuspid socket to cuspid socket, the soft tissues were laid back, the projecting portion of the alveolar process was removed, and the gum put back in shape.

*Dr. Clapp.*—Had the teeth been long extracted?

*Dr. Brackett.*—The teeth had not been very long extracted.

*Dr. Fillebrown.*—They had been extracted long enough for the cavities of the sockets to be obliterated?

*Dr. Brackett.*—Not wholly. The operation of extraction—the original operation, I should suppose from the appearance of the case—had not been very many weeks before the operation on the process.

*Dr. Fillebrown.*—Perhaps if the expression were used that the periosteum was removed and the tissue lifted up, it might be applied to the palatal side, but in this case the lingual surface was lifted up, and then the anterior process was removed.

I had some knowledge of the case by correspondence beforehand, and so speak of it somewhat familiarly. That is the method that I have pursued in a number of cases. One was a case of the under jaw where the process had been too prominent. Just open the gum, break off the process, and let the gum lie back, and it heals down and makes a perfect bearing on an artificial denture.

The motion is made and seconded that the essayists of the evening receive the thanks of the Academy for their attendance and interesting papers this evening, and that they be requested to furnish a copy for publication.

So voted.

Adjourned.

CHARLES H. TAFT, D.M.D.,
*Editor American Academy of Dental Science.*

